# Neurological complications of *Orthopoxvirus* infections: neurotropism and neurovirulence

**DOI:** 10.1093/brain/awaf181

**Published:** 2025-05-15

**Authors:** Hajar Miranzadeh Mahabadi, Ryan S Noyce, David H Evans, Christopher Power

**Affiliations:** Department of Medicine, University of Alberta, Edmonton, AB, Canada T5N 2S2; Department of Medical Microbiology and Immunology, University of Alberta, Edmonton, AB, Canada T5N 2S2; The Li Ka Shing Institute of Virology, University of Alberta, Edmonton, AB, Canada T5N 2S2; Department of Medical Microbiology and Immunology, University of Alberta, Edmonton, AB, Canada T5N 2S2; The Li Ka Shing Institute of Virology, University of Alberta, Edmonton, AB, Canada T5N 2S2; Department of Medicine, University of Alberta, Edmonton, AB, Canada T5N 2S2; Department of Medical Microbiology and Immunology, University of Alberta, Edmonton, AB, Canada T5N 2S2

**Keywords:** monkeypox virus, variola virus, vaccinia virus, nervous system

## Abstract

With the declaration of monkeypox virus (MPXV) infection as a global health emergency in 2022 by the World Health Organization and its ongoing presence, orthopoxviruses have garnered increasing attention, including their capacity to cause neurological disease. Indeed, the mpox syndrome caused by MPXV infection is recapitulated in humans for several other orthopoxviruses, including variola (VARV, the cause of smallpox), vaccinia (VACV), camelpox (CMPX) and cowpox (CPXV) viruses, albeit with variable disease severities. In addition to prototypic signs and symptoms of *Orthopoxvirus* infections, such as fever, swollen lymph nodes, malaise and skin lesions, MPXV-infected individuals also develop neurological syndromes such as headaches, myalgias, seizures, altered consciousness and encephalopathy/encephalitis. MRI of the brains of MPXV-infected persons can display hyperintensities consistent with brain oedema. Pleocytosis has also been reported in the CSF from persons with MPXV infections, implying active infection of the CNS. Of note, newborn rodents, or animals with severe combined immune deficiency, were found to be susceptible to MPXV infection with evidence that the virus can cross the blood–brain barrier. In the present review, we highlight the current understanding of *Orthopoxvirus* neuropathogenesis together with germane diagnostic and therapeutic considerations.

## Teaser

Orthopoxviruses—including mpox and smallpox—cause symptoms such as fever, swollen lymph nodes, and skin lesions, but can also lead to neurological complications including headaches, seizures, altered consciousness, and encephalitis. Miranzadeh Mahabadi *et al.* review current understanding of *Orthopoxvirus* neuropathogenesis.

## Introduction

**Figure 1:**
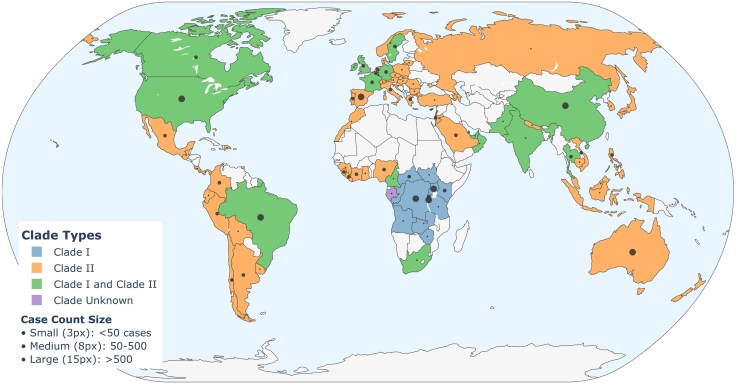
**Global geographic distribution and incidence of new MPXV infections**. Monkeypox virus (MPXV) infection is a global challenge that is apparent on all continents in a clade-specific manner with variable incident rates depending on the geographic location (data from January 2024 to March 2025).

The re-emergence of the mpox clinical syndrome, caused by monkeypox virus (MPXV) infection in 2022 in Western Europe and subsequently in North America, led to the declaration by the World Health Organization of mpox as a global health emergency on two separate occasions in the past few years.^1^ While the incidence rates of mpox have declined subsequently in high-income countries, it remains a serious health issue in low- and middle-income countries (Fig. 1),^2^ especially among children in Central and West Africa.^3^ Indeed, there has been a concurrent growing interest in poxvirus neurotropism and neurovirulence because of a subset of persons infected by MPXV exhibiting multiple neurological signs and symptoms. Nonetheless, other viruses belonging to the Poxviridae family are also known to infect the nervous system and cause neurological disease. Poxviruses are large, enveloped, double-stranded DNA viruses with the unique ability to replicate entirely within the cytoplasm of infected cells.^4^ This family comprises multiple genera, including the *Orthopoxvirus*, comprising several human pathogenic species such as variola virus (VARV), which is the aetiologic agent for smallpox, vaccinia virus (VACV), cowpox virus (CPXV), camelpox virus (CMLV) and MPXV (Table 1).^5,6^ Orthopoxviruses are enveloped double-stranded DNA viruses that range in genome size from 170 kb to 250 kb.^7^ Viral entry of a host cell usually requires binding to glycoaminoglycans followed by endocytosis, fusion and cytoplasmic replication. Poxviruses are released by budding of virus particles and/or cell lysis. Although smallpox was declared eradicated in 1980 following a global vaccination effort,^8^ other orthopoxviruses, notably MPXV, have emerged as zoonotic threats, with recent outbreaks reported beyond their historical endemic regions in Central and West Africa.^5,9-16^

**Table 1:** Human *Orthopoxvirus* infections and their biological features

Virus	Endemic regions	Primary hosts	Reservoir hosts	Vaccine availability	Cross-immunity	Severity spectrum	Modes of transmission	Experimental models
Monkeypox (MPXV)	Central/West Africa	Primates, rodents	Wild rodents, primates	Yes (vaccinia-based)	Cross-reactive with other orthopoxviruses	Mild to severe; rare CNS effects	Close contact, droplets	Mice, prairie dogs, non-human primates
Smallpox (VARV)	Global (eradicated)	Humans only	None	Eradication vaccine	Cross-reactive with other orthopoxviruses	Severe; significant CNS cases historically	Close contact, fomites	Historical data
Vaccinia (VACV)	Laboratory use	Humans (vaccine-related)	None	Yes	Cross-reactive with other orthopoxviruses	Mild; primarily self-limiting	Direct contact, injection	Mice, rabbit, non-human primates
Cowpox (CPXV)	Europe, Russia, Asia	Wild rodents and domestic animals	Wild rodents	Yes	Cross-reactive with other orthopoxviruses	Mild to severe	Close contact, droplets	Mice, non-human primates
Camelpox (CMLV)	Africa, Middle East, Central Asia	Camel and llamas	Camel	Yes	Cross-reactive with other orthopoxviruses	Mild localized infections to severe systemic disease	Close contact, droplets	Mice, camel

**Figure 2:**
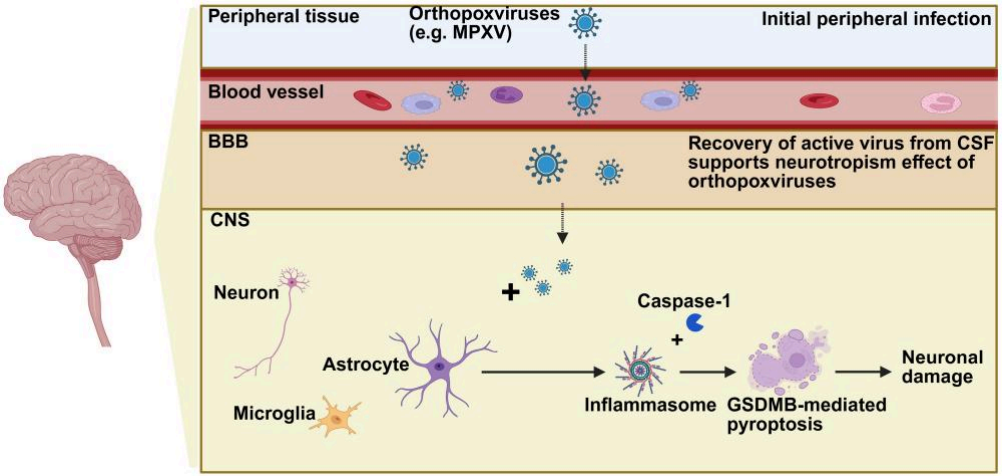
**Contemporary understanding of *Orthopoxvirus* infections in the human CNS**. Neuroinvasion may occur by infected leucocytes trafficking across the blood–brain barrier (BBB) or by direct infection via brain endothelia. Astrocytes are most permissive to infection by monkeypox (MPXV), resulting in inflammatory cell death, termed pyroptosis, which may contribute to neurovirulence through cleavage of gasdermin B (GSDMB) and ensuing cell lysis with adjacent neuronal damage. To date the cell tropism and mechanisms of neurovirulence for other orthopoxviruses remains unknown. Created in BioRender. Power, C. (2025) https://BioRender.com/w95s574.

As of October 2024, more than 100 000 mpox cases have been reported in 122 total countries (Fig. 1), including 115 countries where mpox was not previously reported.^17^ Most mpox cases present with painful mucosal and deep-seated cutaneous lesions, which are typically localized and generally resolve within a few weeks. However, some cases involve more severe symptoms and disseminated disease, which have been associated with increased mortality risk. Although poxviruses have not been regarded as classical neurotropic viruses like herpesviruses, flaviviruses, retroviruses or enteroviruses,^18,19^ there is increasing recognition that orthopoxviruses can infect and cause significant damage to the nervous system (Table 2).^20-22^ Emerging evidence, particularly from the 2022 mpox outbreak, underscores the neurotropic and neurovirulent properties of poxviruses, raising concerns regarding their impact on both the central and peripheral nervous systems. In this review, we discuss the neurotropic and neurovirulent characteristics of orthopoxviruses, with a primary focus on MPXV. We also review the clinical and demographic features associated with neurological manifestations together with the neuropathogenic mechanisms that drive CNS and PNS syndromes (Fig. 2).

**Table 2:** Orthopoxviruses with associated neurological complications in humans

Virus	Animal to human transmission	Human to human transmission	Systemic signs and symptoms	Neurotropism and neurovirulence
Monkeypox (MPXV)	Common (e.g. rodents, primates)	Limited (e.g. close contact)	Fever, rash, lymphadenopathy, myalgia	Neurological complications (encephalitis, seizures)
Smallpox (VARV)	No	Yes (aerosol, direct contact)	Fever, rash, systemic infection	Rare (encephalitis in severe cases)
Vaccinia (VACV)	Rare (laboratory animals)	Rare (e.g. vaccine contact)	Localized rash, fever, fatigue	Rare (encephalitis, myelitis in immunocompromised), Strain dependent
Cowpox (CPXV)	Common (e.g. cats, rodents)	Not reported	Localized lesions, fever	Encephalitis reported in some cases
Camelpox (CMXV)	Rare (close contact with infected animals)	Not reported	Fever, nasal discharge, and lesions	Not reported
Ahkmeta	Rare (contact with infected animals)	Not reported	Cutaneious lesions	Not reported

## Human *Orthopoxvirus* infections

### Variola virus

The variola virus (VARV), which exclusively infects humans and has no natural animal reservoir, spreads primarily through the respiratory tract with infection of the oropharyngeal or respiratory mucosa (Table 1).^23^ It can also transmit via skin contact, bodily fluids or pock material of infected persons. After an incubation period of 7–19 days, patients experience fever, malaise and headaches, followed by a rash that progresses through stages, forming deep-seated, synchronous lesions, 0.5–1 cm in size.^24^ Smallpox first became endemic in regions like India and Egypt around 2000 Bc and subsequently spread. In the late 1700s, Edward Jenner suspected that exposure to CPXV derived from horsepox could be used as a preventative against smallpox infection in humans.^25-27^ Later, the repeated passage of viral material by Jenner and others indeed contributed to the emergence of a common ancestor of the VACV.^28,29^ By the 1940s, smallpox was nearly eradicated in countries like the USA, but in 1967, there were still millions of cases worldwide, prompting a global vaccination effort using VACV that led to the eradication of smallpox in 1979. There were two major variants of smallpox, variola major and variola minor, distinguished by their clinical severity and geographic distribution. Variola major, the more severe form, had a mortality rate ranging from 5% to 40%,^30^ primarily affecting the Indian subcontinent, Africa and Asia. Variola minor, a milder form with a mortality rate of 0.1%–5%,^30^ was more commonly seen in eastern Africa and the Americas, causing fewer lesions and significantly lower fatality rates.^24^ The strains eradicated in North and South America were mostly variola minor.^31^

### Monkeypox virus

MPXV causes a syndrome in humans similar to smallpox,^32^ with mortality rates reaching up to 10% in some circumstances.^33^ Mpox presents with clinical symptoms, comparable but less severe than smallpox, including fever and vesicular-pustular skin lesions. Mpox lesions in the anogenital area often develop asynchronously, followed by the trunk, limbs, face and palms/soles.^34^ Lesions tend to appear at the same time and progress through several stages (macular, papular, vesicular, and pustular) before scabbing over and desquamating.^34^ Unlike smallpox, mpox is often accompanied by lymphadenopathy, resulting in swollen lymph nodes.^35^ The disease typically begins with a prodromal phase characterized by fever, headache and lymphadenopathy, followed by a distinctive rash that evolves through several stages. Recognizing these manifestations is crucial for timely diagnosis and management.^36-39^

MPXV was first identified as pox-like outbreaks in two groups of cynomolgus macaques (*Macaca fascicularis*) at the State Serum Institute in Copenhagen, Denmark in 1958.^40-42^ The first human recorded case of mpox was in a 9-year-old boy in 1970 from the Democratic Republic of the Congo.^43^ MPXV can be transmitted from infected animals such as non-human primates (NHPs), Gambian pouched rats and squirrels to humans. Animal-to-human transmission occurs through direct contact with animals’ body fluids, bites, scratches, or via the handling of infected animals.^44,45^ Human-to-human transmission, while initially regarded as inefficient, has become more prevalent, with the number of human-to-human transmission cases increasing from 29.6% in the 1980s to 73% by 1997.^46^ Since the cessation of smallpox vaccinations in 1980, the incidence of mpox has increased substantially, especially in Central Africa, where human mpox cases have escalated by 20-fold.^25,47-50^ Human-to-human transmission occurs mainly through close contact with infected individuals, including healthcare workers, sexual partners and household members, via respiratory droplets, contaminated materials and bodily fluids like lesions and mucosal secretions.^51^ During the 2022 outbreak, mpox predominantly affected men, particularly those who have sex with men.^52,53^

MPXV has two distinct clades: Clade I (Central African), primarily found in Central Africa and associated with greater virulence, and Clade II (West African), which includes the West African strains responsible for less severe outbreaks. Phylogenetic analysis confirms the existence of these two clades, with Clade I generally being more pathogenic than Clade II.^54-57^ In 2022, there was a notable global surge in mpox cases caused by Clade IIb MPXV, with outbreaks spreading rapidly across Europe, the Americas and other continents.^1^ A more recent Clade Ib outbreak prompted the WHO to declare a second public health emergency of international concern in August 2024. This sub-clade appears to affect individuals under 15 years old predominantly. The virus can be diagnosed using genetic, phenotypic, and immunologic methods, including PCR, immunohistochemistry and electron microscopy.^58-61^ While MPXV's neuroinvasive potential is less well-documented than other poxviruses, recent studies suggest it can cause CNS complications, which may result in long-lasting neurological deficits, highlighting the need for further research into its effects on the nervous system (see later).

### Cowpox virus

CPXV is another zoonotic disease, which has a broad host range and infects a wide variety of domestic and wild animals.^62-64^ At least five distinct clusters have been identified through phylogenetic analysis.^65^ Human cases are predominantly reported in Europe, especially in rural areas where human-animal interactions are more frequent. Several cases of CPXV transmission from cats to humans^66-69^ and from rats to humans have been documented.^70,71^ There has also been an instance of CPXV transmission from a brown rat (*Rattus norvegicus*) to an Asian elephant (*Elephas maximus*) and subsequently to a human.^72^ The virus generally causes localized skin lesions in humans, usually on the hands or face, which may progress to vesicular or ulcerative forms. Systemic symptoms often appear when the skin lesion becomes noticeable, including fever, malaise, headache and occasionally vomiting.^73^ While CPXV is relatively low in pathogenicity for healthy individuals, infection can be severe or even fatal in immunocompromised individuals^74,75^ or pregnant women, posing a particular risk to the fetus.^76^ Immunocompromised patients may experience widespread lesions or systemic complications, including pneumonia and septicaemia. Furthermore, there is concern about the virus's potential to cause more severe disease in populations with compromised immunity due to conditions like HIV, chemotherapy exposure or organ transplants.^77^ A definitive diagnosis of CPXV requires laboratory tests, including cultured virus, electron microscopy, PCR and immunohistochemical analysis of clinical samples. These techniques are essential for confirming cowpox infection. Person-to-person transmission has not been documented. CPXV is primarily transmitted through direct contact with infected animals^67,78^ or indirect contact with contaminated surfaces^79^ and respiratory droplets.^80^ Smallpox vaccination significantly impacts the immune response to CPXV, as all orthopoxviruses share cross-reacting antigens.^81^

### Vaccinia virus

Recently, there has been a significant rise in *Orthopoxvirus* infections caused by zoonotic VACV, which includes buffalopox, affecting farm animals like cows, horses and buffaloes, as well as humans in countries such as India, Brazil and Colombia.^82-90^ Clinical features associated with VACV infection in humans are characterized by skin lesions, accompanied by systemic symptoms such as fever, lymphadenopathy and headache.^91-94^ These clinical features arise from occupational exposure, particularly through handling infected dairy cattle. VACV was specifically chosen as the primary vaccine strain against VARV due to its ability to generate strong cross-protective immunity. Although genetically distinct, both viruses share key antigenic similarities that enable the immune system to recognize and neutralize VARV following VACV immunization.^95,96^ Determining the origin of VACV has been challenging. Jenner's smallpox vaccine was originally thought to be derived from CPXV, but it is now known that VACV, used in the vaccine, is more closely related to horsepox virus.^27,97^ The natural reservoir of VACV remains uncertain, with theories suggesting it emerged from precursor viruses passed through animal skin during the preparation of smallpox vaccines. Person-to-person transmission has been documented, potentially through direct contact or shared environments.^98^ The diagnosis of VACV infection is typically based on clinical presentation and can be confirmed through serological and molecular techniques. These methods include the detection of the viral genome or isolation of the virus from clinical samples.^91^

### Camelpox virus

CMLV infections in dromedary (*Camelus dromedarius*) and Bactrian camels (*C. bactrianus*) can lead to symptoms such as fever, nasal discharge and pox lesions on various body parts, including the head, neck, limbs and genital areas.^99-101^ Infected camels, particularly those over 2 years old, usually experience low morbidity (around 1.1%), but fatality rates can range from 12% to 25% in severe cases. Histopathological findings often reveal dermatitis with ballooning keratinocytes, and in some cases, bronchitis or pneumonia can develop, leading to death.^100,101^ Human cases of CMLV have been reported in association with infected camels. In 2009, an outbreak in Rajasthan, India, affected three people who developed fever, pruritis, erythema and pox-like lesions on their hands, which progressed to vesicles and crusted over within 15 days.^102^ Viral DNA was detected in one individual and isolated from 14 camels linked to the outbreak.^99^ Another outbreak in eastern Sudan in 2014 showed similar transmission from camels to humans, with symptoms such as fever, malaise, and skin nodules that developed after contact with infected camels. In this instance, viral DNA was found in two camels and three of four humans tested.^102^ Of note, other more genetically divergent orthopoxviruses such as raccoonpox, borealpox and Ahkmeta viruses have been associated with human disease and warrant attention because of their potential xenotropic properties.

## Neurological complications of *Orthopoxvirus* infections

Neurological complications associated with *Orthopoxvirus* infections (Table 2), although less common than the primary cutaneous manifestations,^103^ represent a significant aspect of disease presentation.^22,104-107^ Symptoms including headache, myalgia, fatigue, photophobia and mood disturbances are frequently reported with *Orthopoxvirus* infections (Table 2), particularly mpox.^20,22,104,108-110^ More severe neurological manifestations, though rare, include encephalopathy/encephalitis, seizures, transverse myelitis and acute demyelinating encephalomyelitis (ADEM), which have been observed in persons with mpox and VACV infections.^20,22,104,111^ Neuropathological assessment of persons with smallpox-associated neurological findings disclosed acute perivenular demyelination.^111^ Smallpox-associated encephalitis showed a prevalence of 1/500 patients with variola major and 1/2000 patients with variola minor presenting at 6–10 days of illness.^20^ Encephalitis in mpox can lead to severe confusion, seizures or even coma.^112-114^ A case report of a person with mpox described both motor and sensory deficits in the lower limbs with altered consciousness.^114^ Karin *et al*.^115^ and Sejvar *et al*.^116^ found intrathecal antibody production in the CSF of persons with severe mpox-associated encephalopathy, despite negative MPXV PCR results. Although the incidence of encephalopathy in MPXV infections is low, it represents a serious outcome when present. Myelopathy has been reported in some MPXV cases, manifesting as paralysis or sensory deficits^117^; indeed, transverse myelitis has been observed in conjunction with encephalitis and persistent neurocognitive deficits in a patient with mpox, indicative of ADEM, with an accompanying positive PCR results in CSF for MPXV.^118^ Meningitis associated with mpox occurs despite negative MPXV PCR results in CSF.^115^ A rare but notable complication of mpox is Guillain–Barré syndrome, which was documented in a patient with confirmed MPXV infection.^119^ Likewise, Parsonage–Turner syndrome has been reported as a complication of mpox.^120^ Although less well-characterized, neuropsychiatric symptoms such as anxiety, depression and suicidal ideation have been noted in persons with mpox, indicating a broader impact on CNS functions.^106,116,121^ While the neurological manifestations of MPXV are concerning, it is essential to consider that not all infections lead to severe complications. Many cases may present with mild symptoms, underscoring the need for ongoing surveillance and research to better understand the full spectrum of mpox-related neurological syndromes.

Persons with smallpox were reported to exhibit delirium or encephalopathy during the febrile stage, and febrile seizures can occur in young children.^106^ Encephalopathy/encephalitis may be evident in some smallpox cases with CSF showing elevated pressure and mild lymphocytic pleocytosis, though the virus is typically not cultured from the CSF. Post-mortem brain neuropathological analysis of patients with severe smallpox encephalitis confirmed demyelination.^122^ A case series of smallpox vaccinated subjects described acute perivascular myelinoclasis, or ADEM, as well as cases of transverse myelitis^123^ with concurrent optic neuritis, following smallpox vaccination. Patients with transverse myelitis often present with more motor than sensory symptoms, with pathology indicating greater involvement of grey matter compared to white matter.^20^

## *Orthopoxvirus* neurotropism and neurovirulence

Although poxviruses are not typically neurotropic, evidence shows they can invade and impact the nervous system. Understanding the determinants of neurological diseases is crucial for elucidating the pathogenesis of poxvirus-related neuroinvasion and the associated neurological outcomes. Infection is mediated by viral proteins interacting with host glycosaminoglycans, initiating cellular endocytosis and enabling viral entry. In poxvirus infections, two types of virions—intracellular mature virus and extracellular enveloped virus—are involved in spreading the infection within the host.^36,124^ This mechanism underscores the virus's complex transmission dynamics and its potential to cause further health complications, including neurological damage. Results from rodent studies suggest that MPXV can cross the BBB,^125-130^ confirming the neuroinvasive capacity of the virus. Experimental MPXV infection via intranasal and intraperitoneal routes caused disease and death in ground squirrels within 9 days, with necropsy revealing high MPXV titres in brain tissue.^131^ MPXV DNA was detected by PCR, bioluminescence imaging or plaque assay in brain tissues from multiple experimental animal models.^126,127,129,130,132-135^ Moreover, pathological analyses and detection of MPXV in autopsied brain tissues were conducted on a renal transplant patient with fatal mpox.^136^ Based on these clinical and experimental findings, MPXV seemingly infects the brain in both humans and animals^136,137^; however, further research is needed to determine its neuroinvasive and neurotropism mechanisms. For example, identifying the transmission pathways of MPXV to the CNS is crucial: animal studies imply that MPXV may reach the brain through direct invasion via the olfactory epithelium or indirectly via infected immune cells such as monocytes/macrophages traversing the BBB (e.g. a Trojan Horse-like mechanism). Following intranasal exposure, MPXV replicates in the nasal mucosa and rapidly invades the brain parenchyma.^130^ Alternatively, during systemic infection, infected monocytes may cross the BBB either directly or through inflammatory-induced BBB disruption.^104^ These findings imply that both direct and blood-mediated routes enable MPXV neuroinvasion, often following systemic replication. MPXV-Zaire 79 antigens have been detected in circulating monocytes of NHPs after intravenous injection.^138^ Similarly, subcutaneous injection of MPXV in cynomolgus macaques resulted in a marked increase in viral particles within alveolar and mediastinal lymph node macrophages, suggesting robust viral replication in these cells, as observed by electron microscopy.^139,140^ Although the exact neural receptors engaged by MPXV remain unclear, studies suggest that MPXV preferentially infects human astrocytes, followed by microglia and oligodendrocyte-like cells, activating inflammatory response and pyroptosis, particularly in astrocytes (Fig. 2).^141,142^ Of historical interest, the mouse *Orthopoxvirus*, ectromelia virus, infects the rodent brain^143^ principally in glial cells, causing neuronal death associated with caspase-1 and -3 activation, reminiscent of recent reports indicating inflammasome activation with neuronal death in the setting of virus infection of glial cells.^144^ Moreover, various studies indicate the viral infections can lead to significant changes in micro RNA (miRNA) expression within the CNS. MPXV infection may alter host miRNA expression, affecting neuronal survival, neuroinflammation and BBB integrity. Identifying specific miRNAs involved in MPXV neuropathogenesis could enhance understanding of host-virus interactions and provide potential biomarkers or therapeutic targets for neurological complications.^145^ While treatment for mpox remains to be verified by randomized clinical trials, two antiviral agents—tecovirimat and brincidofovir—have been proposed as potential therapies. Tecovirimat inhibits viral envelope formation and release, whereas brincidofovir, approved for smallpox, targets the viral DNA polymerase and is currently used off-label for mpox. However, recent clinical trials with tecovirimat for the treatment of mpox have shown no significant benefit over placebo.^146-150^ PA104 inhibited viral spread, while cidofovir targets viral DNA polymerase but is limited by nephrotoxicity.^151,152^ Similarly, vaccinia immune globulin intravenously-delivered has been used in mpox and VARV-associated disease, described in case reports and without randomized clinical trials.^153^ Monoclonal antibodies targeting viral surface proteins (e.g. B5R, A33R, H3L) aim to neutralize virus spread.^154^ Antisense oligonucleotides can silence essential viral genes, including those for DNA polymerase and transcription factors, showing promise in preclinical studies.^155^ Additionally, host-directed therapies that modulate immune responses or block viral entry are under exploration.^156^ Ongoing research aims to develop new treatments for *Orthopoxvirus* re-emergence, but further studies are needed to confirm their effectiveness.

While mpox is the current focus of concern due to recent outbreaks, other orthopoxviruses such as VACV, VARV and CPXV also demonstrate neurotropic potential.^157^ Historical and experimental evidence suggests these viruses can invade and damage the nervous system under certain conditions. In some post-vaccinal encephalitis (PVE) cases, VACV was recovered from CSF and autopsied brain tissues, suggesting virus penetration into the brain through the BBB.^157,158^ Neurovirulence in different VACV strains is a genetically determined trait influenced by specific genomic features. The selection of strains like VACV strain Western Reserve (WR) and Clone-3 (CL3) through passage in mouse brains highlights the variability in neurovirulence among different strains, which can lead to varying degrees of risk in human populations. This adverse outcome may reflect a viral genetic predisposition to neurovirulence, the immune response modulation by different strains, and has implications for vaccine safety.^159^ Both the neurovirulent WR and the vaccine strain VACV-Lister replicated in differentiated endothelial cells, although replication was limited. VACV-WR infected and spread between endothelial cells, causing cell lysis, tight junction disruption and increased BBB permeability, enabling potential CNS entry.^157^ In a mouse models simulating viraemia, VACV-WR replicated significantly in the brain, confirming its neurovirulence,^159^ while VACV-Lister showed minimal brain presence, likely due to low replication. Multigene deletions in related viruses like rabbitpox further highlight genetic influences on neurovirulence.^160^

Neuroinvasion by VACV-WR is strain-dependent. VACV-WR can infect and replicate in choroid plexus epithelial cells, potentially disrupting the blood–CSF barrier and contributing to neuroinflammation. Previous studies suggest that the choroid plexus might serve as a site for viral entry and pathogenicity, particularly during encephalitis following vaccination.^157,161-164^ It is important to note that the WR strain was never used as a human vaccine; it was deliberately propagated in mice to create a laboratory strain. The use of intracerebral inoculation in this process may have inadvertently selected for neurovirulent phenotypes. Subsequent applications of VACV-WR in humans, such as in oncotherapy, have employed attenuated derivatives of this strain.

This neuroinvasion underscores VACV's potential as a neurotropic virus, albeit with low frequency in clinical settings.^165^ VACV can also infect and replicate in several types of neural cells, showing permissiveness in astrocytes,^141,166^ neurons^167-169^ and microglia.^166^ Astrocytes are particularly susceptible, allowing VACV replication and subsequent spread within the CNS. Neurons are also permissive to VACV infection, which can lead to direct neuronal damage and death with accompanying neuroinflammation, contributing to neurovirulence. Additionally, VACV has been used as a vector to deliver transgenes into neuronal cells.^170^ Microglia, the resident immune cells of the CNS, can be infected with VACV as well, during which they may play dual roles: (i) facilitating viral replication; and (ii) triggering host inflammatory responses. The VACV’s ability to infect multiple neural cell types underscores its neurotropic nature and the potential for widespread neural impact in susceptible hosts.

CPXV exhibits neurotropism and associated research indicates that the infection can lead to neurological complications, as evidenced by infected persons in whom infection caused severe inflammatory responses with associated neurological symptoms following transmission from infected animals such as cat to human.^171^ While human cases of cowpox with neurological involvement are rare, they offer further insight into the broad neurotropic potential of orthopoxviruses.

It also bears mention that substantial neurological complications have been reported with the older forms of the smallpox vaccine. The complications associated with different smallpox vaccinations is clearly dependent on the individual viral strain.^172^ Historical data indicate that VACV-induced PVE as well as encephalomyelitis (PVEM) occur in both humans and animals in exceptional conditions (e.g. immunosuppression, co-infection with pathogen agents, or with other comorbid conditions).^123,173-177^ These syndromes often started with signs of increased intracranial pressure and frequently progressed to stupor, seizures, paralysis, mental status alterations, sometimes leading to coma.^107^ In PVEM and PVE cases, CNS lesions were found in the cerebrum, medulla and spinal cord at 7–14 days after vaccination.^20,173,178^ CSF analyses showed raised intracranial pressure, while PVEM cases specifically displayed elevated CSF protein levels along with increased monocytes and lymphocytes. Other neurologic events linked to the VACV include Guillain–Barré syndrome, cranial neuropathies (including Bell's palsy) and poliomyelitis-like syndrome.^179^

## Future perspectives

While MPXV, VARV and VACV are recognized for their ability to cause neurological complications, the other orthopoxviruses that are known to infect humans seem to be less neuropathogenic. Nonetheless, this might reflect reduced awareness of the potential for neurological disease as a complication of *Orthopoxvirus* infections. The potential for future cross-species infections is highly plausible, given the burgeoning concerns regarding increased human-animal interactions, climate change, medical interventions and viral evolution, underscoring the imperatives for further surveillance and research to assess emerging risks. At the same time, as human-animal interactions evolve, new orthopoxviral (e.g. raccoonpox, borealpox and Akhmeta) infections also require ongoing scrutiny. Diagnostic options, including analyses of viral genome by PCR or immune responses in blood and CSF, are of paramount importance, together with neuroimaging, in establishing the diagnosis of an *Orthopoxvirus*-related neurological complication. Studies of viral neuropathogenesis using *ex vivo* human neural cells as well as permissive animal models, such as humanized mice used in studies of variola,^180^ will advance the understanding of *Orthopoxvirus* mechanisms of disease, leading to the development of rational therapies in the future.
